# Expanding Horizons of Diagnostic Imaging

**Published:** 1988-11

**Authors:** P. N. T. Wells

**Affiliations:** Department of Medical Physics, Bristol General Hospital


### Expanding Horizons of Diagnostic Imaging

**Professor P. N. T. Wells**
Department of Medical Physics, Bristol General Hospital

The past 25 years have seen remarkable developments in body imaging. These have occurred as scientists have come up with new methods of producing body images which doctors have taken up, rather than doctors identifying a need which scientists have responded to. Medical imaging goes back to 1895 and to Wilhelm Konrad Roentgen, the physicist who discovered X-rays. In 1898 the Skiagraphic Department opened at the BRI, followed by another, 3 years later, at the BGH.

Until 25 years ago X-rays were the only means of imaging the inside of the body. It has recently been decided to screen all women between the ages of 50 and 64 for breast cancer and so Professor Wells chose **mammography** for his example of radiology. The difficulties were illustrated by a case in which a tumour had been shown on mammography but the surgeon at operation had failed to find it. 4 years later another mammogram showed that the earlier tiny tumour had developed.

The **image intensifier** is a device by which an X-ray can be demonstrated on a TV screen, it can be fed through an analogue processing system with additional brightness or through a computer system in which images are stored, this kind of technique enables pictures to be taken before and after injection of contrast medium and then by subtracting one from the other show up contrast medium that would not otherwise be visible. This sort of technique is especially useful in the dilation of arterial strictures by balloon catheters. If the catheter cannot be made to pass the stricture a laser can be used to heat the tip of a catheter to drive it more easily through the obstruction.

In 1974 occurred the development of **digital computed tomography**, this can make cross sectional images through the body and we can think of the patient as being made up of a series of slices. This is done with an X-ray tube and a detector, the two are translated together across the patient to collect the X-rays as they are attenuated travelling along different paths through the patient, then they are rotated through a small angle to collect a whole family of these transmission profiles and from that information it is possible to reconstruct an image of the cross section through the body. This was the method used by Godfrey Hounsfield and for which he was awarded the Nobel Prize. Original scanners had a time of two minutes which has now been reduced to about a second for a conventional CT scanner. This is not fast enough to freeze the motion of the heart and so a new kind of CT scanner has been produced in which there are no moving parts but the X-ray source is moved around the patient electronically by deviating an electron beam onto the target within a sealed tube, like a giant television tube with a hole in the middle, inside which the patient lies. This reduces the scanning speed to about 10 milliseconds.

Another method of imaging using ionising radiation, developed in the mid 1950s, is the **gamma camera** which depends on radionuclides. These are administered to the patient and are taken up selectively by different parts of the body and emit gamma rays which will produce an image in the camera positioned over the patient. These images can also be made in a dynamic way for example to show the contracting ventricles of the heart and thus demonstrate an infarct.

There have also been very important developments on the pharmaceutical side particularly involving monoclonal antibodies which allow the radionuclides to be targeted very selectively to different parts of the body so that images can be not only structural but also functional giving information about the metabolism of tissues and their immune response.

Another type of imaging useful in research is positron emission transaxial tomography which uses very short lived radionuclides such as carbon and fluorine, these can be incorporated directly into biological molecules so that they can take part in the metabolism which is being studied. It produces very powerful images but a disadvantage is that a cyclotron on-site is also needed to produce these short lived radionuclides and the opportunities for using it are rather few.

Other techniques which do not use ionising radiations have advantages for this reason and are also complimentary. Ultrasound scanning is one of them. A probe shines through the patient producing on a screen a series of echoes along the ultrasonic beam, the beam is moved around the patient, the echoes appear in a new position and a cross sectional image can be built up of the patient in that plane. These images can be provided in grey scale and now in colour showing arteries in red and veins in blue according to the direction of blood flow.

**Magnetic resonance** imaging as used at the moment is a technique for imaging the proton distribution and the behaviour of protons within the body. Water, a constituent of every living thing, is fortunately a very good nuclide for study by magnetic resonance. The proton, the nucleus of the hydrogen atom, is spinning and it behaves like a little magnet. It has special properties when placed in a magnetic field, analagous to a spinning top, spinning vertically first and then moves over to its side and processes around at a particular frequency depending upon the properties of the top and of the gravitational field. A proton behaves in the same way. A magnetic field replaces the gravitational field and its frequency of rotation will depend upon its distance from the magnet and give an indication of the position in the body of the proton itself. Where blood is flowing it is possible to pick out blood vessels without the use of contrast medium. It can also give information about the biochemistry of tissue and for example show up ischaemia of the myocardium.

Professor Wells illustrated his talk throughout with superb slides and videos of the techniques described. He ended by saying that we may have had the impression of going through an Aladdin's cave of very expensive toys. However they are not toys and they are not very expensive for the individual patient. What is expensive is the treatment which the diagnostic information obtained by these new techniques makes possible.

